# Beyond flatland: naturalistic three-dimensional stimuli and visual working memory processing

**DOI:** 10.1007/s10055-026-01343-0

**Published:** 2026-03-21

**Authors:** Gilad Schrift, Shachar Lando, Roy Luria, Nitzan Censor

**Affiliations:** 1 Sagol School of Neuroscience, Tel Aviv University, Tel Aviv, 69978 Israel; 2 School of Psychological Sciences, Tel Aviv University, Tel Aviv, 69978 Israel

**Keywords:** Visual working memory, 3D perception, Virtual reality, Change detection, Cognitive load, Depth cues, Ecological validity

## Abstract

Visual working memory (VWM) enables the temporary storage and manipulation of visual information, yet its limited capacity makes it sensitive to the amount and structure of the information it must retain. The vast majority of previous VWM research has used two-dimensional stimuli, while real-world visual perception incorporates depth-related spatial cues which may affect performance. Prior work suggests that depth could enhance VWM performance by enabling perceptual enrichment and better individuation, but may also introduce additional cognitive processing costs and impair performance. Here, we directly tested how dimensionality influences VWM under different memory loads using a virtual reality adaptation of the change detection task, enabling the presentation of ecologically valid, real-world 2D and 3D objects. While accuracy was comparable across stimulus dimensionality, response times for 3D stimuli showed larger increases at higher memory loads. These results suggest that even though 3D stimuli may enrich perceptual input, they introduce processing costs that become more apparent under high memory load, possibly demanding additional neural resources associated with depth processing.

## Introduction

Visual working memory (VWM) is an essential cognitive system which enables individuals to temporarily store and manipulate visual information, thus supporting tasks like navigation, object recognition, and decision-making (Baddeley 1992). VWM maintains an active representation of the visual environment, defined by limited capacity and duration, and interacts closely with other cognitive structures, such as long-term memory and attention (Cowan 2001, Fukuda and Woodman 2017, Bahle et al. 2018). To represent a dynamic visual environment effectively, VWM must continually update its representations (Luria and Vogel 2014, Balaban and Luria 2017). In this process, VWM takes into account not only individual physical features, but also the binding of these features together into complex representations of discrete objects (Chen et al. 2018, Gao et al. 2016, Luria and Vogel 2011). Additionally, spatial location plays a role in organizing and binding features in VWM, with spatial separation of objects enhancing performance and reducing memory interference (Hollingworth 2007, Pertzov and Husain 2014).

**Fig. 1:**
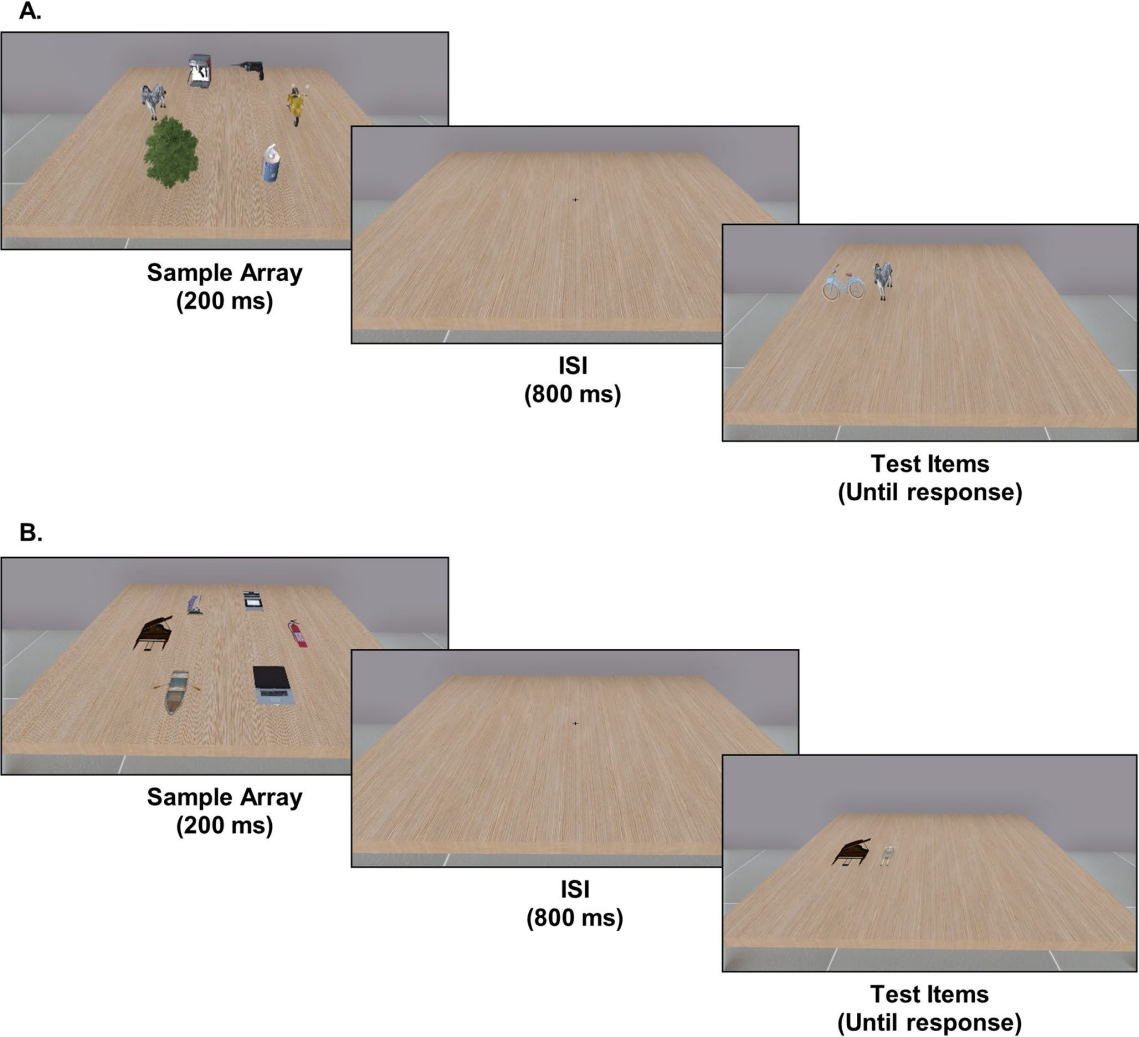
VR implementation of the change detection task, as seen via a stereoscopic HMD. **A** 3D stimuli array. **B** 2D stimuli Array. For both types of stimuli, an array containing 3 or 6 objects appears at fixed locations on the screen for 200ms, then disappears and reappears following an 800ms delay. Upon reappearing, participants determine which of the two items on screen belongs to the sample array. The objects remain until a response is given

While these findings indicate the significance of spatial location in VWM, less is known about the impact of other spatial information, such as depth related cues. Most research has relied on single-plane presentation of 2D stimuli, with recent studies demonstrating that additional depth-related spatial information modulates VWM performance. As will be discussed shortly, these studies report inconclusive evidence. To address this gap, we developed a virtual reality (VR) adaptation of the change detection task using a stereoscopic head-mounted display (HMD), enabling us to test how stimulus dimensionality (2D versus 3D) of ecologically valid, real-world objects interacts with VWM performance under different cognitive loads (Fig. 1). This adaptation builds on recent VR studies, which demonstrated the feasibility of change detection paradigms in immersive settings in replicating the behavioural and electrophysiological features of VWM (Klotzsche et al. 2023, Bassano et al. 2023).

Depth cues were shown to enhance VWM performance when binocular and monocular depth cues were congruent, with items that were perceived as closer to the observer remembered more accurately (Qian et al. 2017). Additionally, binocular depth cues were shown to facilitate visual perception (Railo et al. 2018) and object individuation, particularly under crowded visual conditions (Chunharas et al. 2019). Separation by depth was also found to improve VWM performance, with performance benefits strongest when the number of items shown exceeded individual memory capacity and when items were evenly distributed across depth planes (Sarno et al. 2019). These findings suggest that depth may serve as an organizational “tag” in VWM, supporting perceptual enrichment and enabling better individuation, particularly under high-demand conditions.

However, some evidence indicates that the benefits of depth cues might be constrained by the cognitive demands associated with processing 3D information. Performance in VWM tends to decline under concurrent cognitive load, especially when stimuli are structurally complex or perceptually demanding (Ricker and Vergauwe 2022). Increased memory demands have also been shown to impair perceptual processing, likely due to shared resource limitations (Emrich et al. 2011). Furthermore, increasing the dimensionality or spatial connectivity of stimuli did not lead to enhanced memory performance in a color change detection paradigm, as connected 3D cubes did not improve accuracy compared to unconnected 2D squares (He et al. 2022). These findings suggest that while depth information may provide organizational benefits, it could simultaneously introduce additional processing costs. The mnemonic value of 3D structure in VWM might be dependent on the relevance of depth cues to the task at hand, and structural complexity may not support memory unless it facilitates task-relevant processes like individuation. Notably, most of these findings are based on abstract stimuli, such as rendered shapes with depth cues, which may differ in their cognitive demands from real-world objects.

To address these questions, we leveraged VR HMD, enabling the presentation of ecologically valid, real-world objects within naturalistic 3D contexts. This allowed testing two competing hypotheses: (1) If perceptual enrichment drives the effect, 3D objects should show superior performance compared to 2D objects, with this advantage becoming more pronounced under high memory load conditions; (2) If cognitive load is the primary factor, 3D objects should show impaired performance relative to 2D objects, with greater disadvantage as memory demands increase.

## Methods

### Participants

Forty healthy adults aged 21–35 years (35 females, *M*_*age*_ = 23.6, *SD* = 3.07) completed the study, which was approved by the Tel Aviv University’s Ethics Committee. All participants provided written informed consent to participate in the study, approved having normal or corrected-to-normal vision without wearing glasses, and reported at least 7 h of sleep the night before the experimental session. Participants received monetary compensation or course credit for their participation. Sample size was based on G-power (Faul et al. 2007) calculations of effect size (*η*_p_^2^ = 0.1, power = 0.9) estimated based on the results of a pilot experiment.

### Materials

The task was constructed using Unity engine (Unity Software Inc, San Francisco, CA), rendered using a GeForce RTX3090 graphics card (NVIDIA, Santa Clara, CA), and presented by means of a Pimax 8KX head-mounted display (HMD) with a 140° diagonal field-of-view and a resolution of 3840 × 2160 per-eye. Participants responded using the left/right buttons of a wireless mouse in order to avoid any complexity caused by introduction of a novel control method.

Stimuli consisted of 21 familiar objects from four categories: 6 household objects, 4 vehicles, 6 tools, and 5 natural objects. These categories represent a broad range of real-world objects relevant to everyday experiences, enhancing the ecological validity of the stimuli. Objects within each category were comparable in terms of size and shape complexity while maintaining visual distinctiveness, minimizing potential confounds related to visual or cognitive salience. Each item had both 2D and 3D variants matched for angular size and spatial extent in the VR environment, ensuring that dimensionality effects were not confounded by size differences between presentation formats.

For 2D stimuli, images were captured from a fixed perspective directly aligned with each object’s central axis to minimize depth cues and prevent significant parallax or perspective distortion. Shadows and reflections were removed during rendering, with uniform lighting applied to prevent shading gradients that might suggest depth. A neutral gray background was used throughout.

### Experimental design

The study employed a within-subjects 2 × 2 design, with the factors of Dimension (2D vs. 3D) and Set Size (3 vs. 6). Participants were given verbal and written instructions and completed a series of practice trials to familiarize themselves with the task before the experiment began. Each participant completed two runs of a VR variant of the change detection task, alternating between 2D and 3D stimuli across runs. The stimuli were presented at fixed spatial locations arranged in a circular configuration, forming even distribution across the visual field and avoiding overlaps. To emphasize depth cues for 3D stimuli, they were presented with a slight varying random tilt on their horizontal axis (between − 12.5° and 12.5°), while 2D stimuli were presented at a fixed angle. The order of 2D and 3D runs was counterbalanced across participants to control for potential order effects. Each run consisted of four blocks of 40 trials in which set sizes were mixed, summing to a total of 80 trials per condition. In each trial, participants were briefly shown an array of 3 or 6 objects presented in parallel for 200 ms. Following an 800 ms inter-stimulus interval (ISI), two objects appeared at one of the original locations, one item which was in that location in the sample array and a distractor. Participants were instructed to report, as quickly and accurately as possible, which of the two test objects was part of the sample array. The test objects remained visible until a response was made.

To minimize VR-related discomfort, the experiment was divided into two runs of approximately 10 min each, separated by a mandatory 2-minute break during which participants removed the HMD. The task involved no locomotion in the virtual environment, and all stimuli were static, thereby reducing common triggers of simulator sickness. The display was rendered at the native refresh rate of the headset to prevent latency-related discomfort. At the end of the session, participants were asked to report any symptoms of simulator sickness or fatigue, with no participants indicating experiencing such symptoms.

### Data analysis

Repeated-measures ANOVAs were conducted using the statistical program JASP (Version 0.95.4.0; JASP Team) to evaluate the effects of Set Size (3 vs. 6), Dimension (2D vs. 3D), and their interaction (Set Size × Dimension) on accuracy and response time (RT). Two Participants whose mean accuracy level was below 50% at one or more of the test conditions were excluded from the analysis. Trials deviating in RT more than three standard deviations from each subject’s mean were considered outliers and excluded from the analysis.

## Results

### Accuracy

**Fig. 2:**
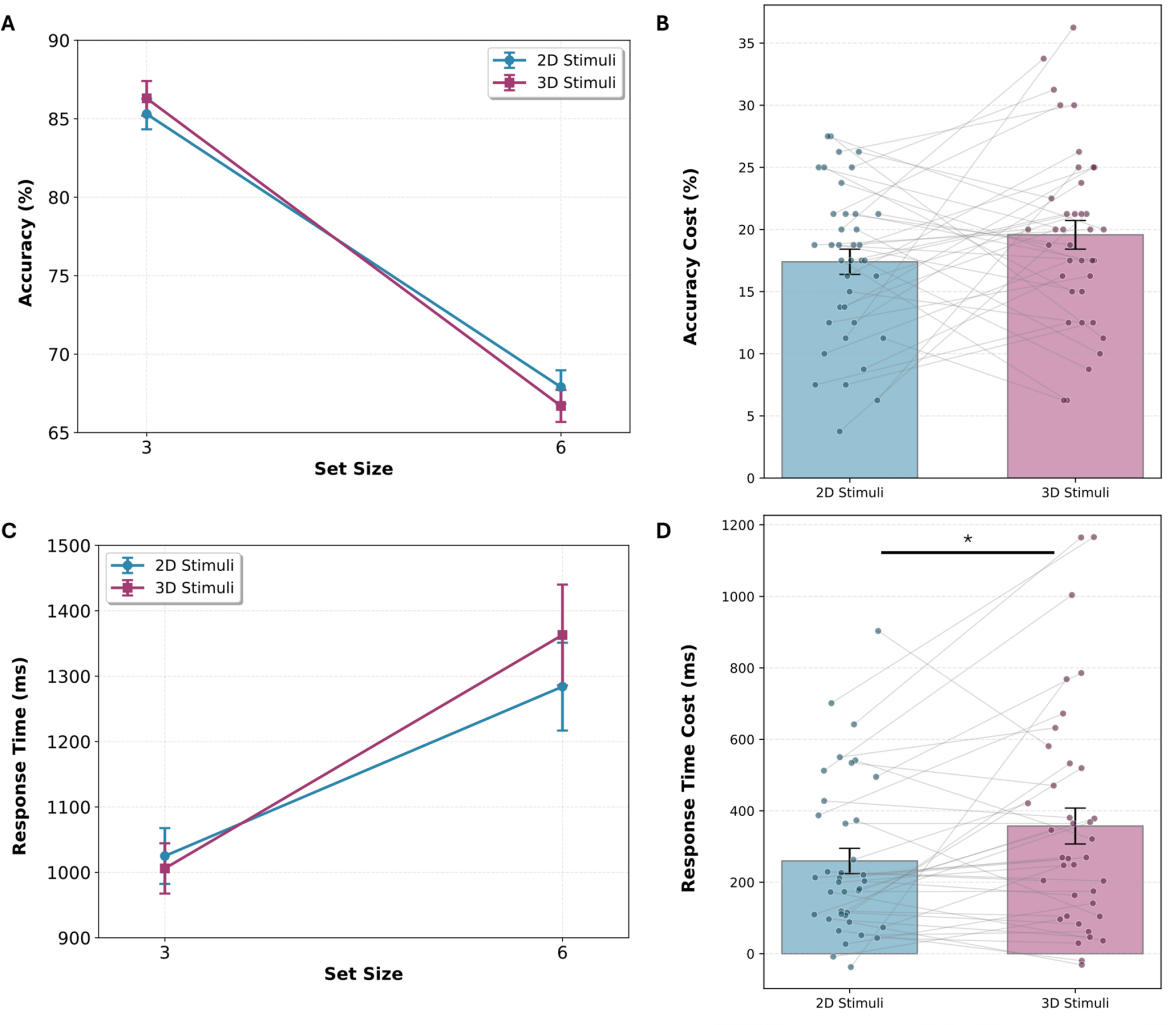
Dimensionality and VWM performance.** A** Accuracy across set sizes for 2D and 3D stimuli. **B** No interaction between Dimension and Set Size for accuracy. **C** Response time across set sizes for 2D and 3D stimuli. **D** Significant interaction between Dimension and Set Size for response time, with a 40% larger RT cost for increasing set size in 3D stimuli. Error bars represent $\:{}_{-}{}^{+}1$ standard error of the mean (S.E.M.), *p < 0.05

A repeated-measures ANOVA revealed a significant main effect of Set Size (F(1, 37) = 522.290, *p* < 0.001, η² = 0.824, η_p_^2^ = 0.934), with accuracy decreasing substantially as set size increased. In the 3D condition, mean accuracy declined from 86.3% ± 1.1% S.E. for 3 objects to 66.7% ± 1.0% for 6 objects (Fig. 2A). The 2D condition showed a similar pattern, with accuracy declining from 85.3% ± 1.0% to 67.9% ± 1.1% (Fig. 2A). There was no significant main effect of Dimension (F(1, 37) = 0.013, *p* = 0.911, η² < 0.001, η_p_^2^ < 0.001). Additionally, there was no significant interaction between Dimension and Set Size (F(1, 37) = 2.23, *p* = 0.143, η² = 0.003, η_p_^2^ = 0.057, Fig. 2B), despite a larger drop in performance associated with increasing the set size in 3D stimuli compared to 2D stimuli. Overall, these results indicate that stimuli dimensionality did not significantly influence accuracy performance.

### Response time

A repeated-measures ANOVA revealed a significant main effect of Set Size (F(1, 37) = 62.291, *p* < 0.001, η² = 0.461, η_p_^2^ = 0.627, Fig. 2C), indicating that participants were slower to respond to larger set sizes. There was no significant main effect of Dimension (F(1, 37) = 0.868, *p* = 0.357, η² = 0.004, η_p_^2^ = 0.023). However, results showed a significant Dimension × Set Size interaction (F(1, 37) = 6.583, *p* = 0.014, η² = 0.012, η_p_^2^ = 0.151, Fig. 2D), indicating that the influence of the set size on response time was modulated by stimulus dimensionality. In the 3D condition, mean response times increased from 1.01 ± 0.04 s for 3 objects to 1.36 ± 0.08 s, while in the 2D condition the mean response time increased from 1.03 ± 0.04 s for 3 objects to 1.28 s ± 0.07 for 6 objects (Fig. 2C). This indicates a 40% larger RT cost (i.e., responses slow down) when set size increased in 3D stimuli (∆RT = 0.35) compared to 2D stimuli (∆RT = 0.25). Thus, while dimensionality alone did not affect RT performance, 3D stimuli caused a steeper increase in response time at larger set sizes compared to 2D stimuli, possibly reflecting increased demands during perception-memory comparison processes (Hyun et al. 2009, Yin et al. 2012).

## Discussion

While VWM typically operates in a three-dimensional environment defined by objects with varying surfaces, depths, and spatial relationships, it is most commonly studied in 2D settings. The current study investigated how the dimensionality of realistic objects interacts with working memory performance using a stereoscopic VR change-detection task. The results show that stimulus dimensionality interacts with memory load, leading to a steeper increase in response time for larger set sizes in 3D compared to 2D stimuli. While the size of this interaction effect was small relative to the expected dominant main effect of set size, the functional cost was substantial, with an increase of 40% in the response time slope for 3D stimuli compared to 2D stimuli. These findings are aligned with the cognitive load hypothesis, suggesting that 3D objects could place additional, scalable cognitive demands compared to 2D counterparts particularly under high memory load conditions, though notably without impairing accuracy.

Traditional 2D VWM research has mostly used stimuli such as colored squares and simple shapes, which may not reflect the full complexity of real-world visual processing demands. This is also true for most studies that manipulated depth. Here, we presented 3D real-world objects to investigate how VWM interacts with ecologically relevant stimulus features. The steeper increase in response times for 3D stimuli under higher memory loads suggests that dimensionality may have prolonged the comparison stage, responsible for contrasting the visual input with the stored memory representation (Hyun et al. 2009, Yin et al. 2012). For example, perceptual individuation or spatial parsing may take longer in a 3D environment, prolonging the decision process without affecting the actual VWM representation (and thus not affecting the change-detection accuracy).

The interaction pattern between dimensionality and memory load may also suggest that 3D processing engages additional computational steps during comparison. When verifying a 3D memory trace, more complex feature-matching operations are required than for flat 2D counterparts, manifesting as a temporal cost that could become a bottleneck when the system approaches its capacity. This aligns with the dual-stream model of visual processing: whereas 2D object recognition is dominated by ventral stream activity (processing object identity), the perception of 3D structures involves the recruitment of the dorsal stream to extract spatial depth from binocular disparity (Welchman 2016, Orban 2011). This also corresponds with previous evidence showing depth information can slow reaction times without impacting accuracy in a 3D mental rotation working memory task (Tang et al. 2022). In addition to the increased reaction times, additional depth cues from 3D stereoscopic stimuli were found to elicit a stronger neural response compared to 2D stimuli, with low-frequency oscillations in the delta and theta ranges playing a pivotal role in modulating attentional allocation (Tang et al. 2022). VR and real-world object presentations were also demonstrated to evoke more naturalistic ERP profiles, along with a greater alpha-band desynchronization than 2D conditions (Kisker et al. 2025, Sagehorn et al. 2024, Kisker et al. 2025). This effect is associated with increased attentional engagement and active sensory processing (Stipacek et al. 2003), and could reflect greater cognitive resources required to encode and maintain complex visual information in depth.

Several points should be considered when discussing the current study. First, real-world 3D processing involves dynamic viewing angles, varying depth relationships, and head movements. These aspects were constrained in our setup for experimental control and validity, but could be addressed in future experiments. For example, a further modified change detection task that makes extensive use of the environment, a possibility which VR HMDs enable (Scarfe and Glennerster 2015). Furthermore, testing a broader range of set sizes (Railo et al. 2018) could shed additional light on the nature of the interaction, such as whether it follows a linear or non-linear pattern or if it emerges when crossing a specific capacity threshold. In addition, our behavioral measures captured only final performance outcomes, leaving the temporal dynamics of the processing unexplored. Electrophysiological recordings could highlight whether the observed 3D RT costs reflect slower comparison and verification stages, distinctions that are crucial for understanding the underlying mechanisms. Finally, although multiple design features were implemented to minimize potential effects of VR-related fatigue (see Methods), those may still influence performance relative to real-life settings. Future work addressing these points could provide a more complete picture of how dimensional complexity affects visual working memory across varied contexts and processing stages, and may also be extended to long-term visual memory (Censor et al. 2016, Kondat et al. 2024, Klorfeld-Auslender et al. 2022).

In summary, our findings reveal that 3D representations may introduce higher time costs under increased cognitive load, consistent with added demands of perceptual comparison. This highlights a dynamic interaction between perceptual richness and efficiency in visual cognition and, in turn, may carry implications for modulating memory performance in naturalistic environments.

## Data Availability

The experimental data and analysis code have been deposited on the Open Science Framework (OSF) and are publicly available as of the date of publication at https://osf.io/hkuys .An executable version of the task is available at https://github.com/Giladsc/Object-Change-Detection-VR .
